# Editorial: Gut Microbiota in the Occurrence, Development and Treatment of Gut-Brain Disorders

**DOI:** 10.3389/fcimb.2021.808454

**Published:** 2021-12-15

**Authors:** Shengjie Li, Jing Wei, Tingtao Chen

**Affiliations:** The Institute of Translational Medicine, Nanchang University, Nanchang, China

**Keywords:** gut microbiota, psychiatric disorders, gut-brain axis, stroke, fecal microbiota transplantation, psychobiotics, prebiotics

Increasingly, it is recognized that symbiotic microorganisms, specifically the microbiota resident in the gut, may influence neurodevelopment and programming of social behaviors among animal species and humanity (Sherwin et al., 2019). Studies have shown that the gut microbiota-derived signals can transfer to the brain and may alter brain function *via* the microbiota-gut-brain axis, a bidirectional communication pathway between the gut and brain (Dinan and Cryan, 2017). Various neurodevelopmental diseases, including autism, depression, anxiety, and Alzheimer’s disease (AD), are correlated with gut microbiota dysbiosis and altered metabolic activities. Observations across preclinical and clinical data elucidate that targeting gut microbiota through dietary or live bacteria interventions can promise beneficial therapeutic effects on the associated behavioral symptoms in psychiatric disorders (Dinan and Cryan, 2017). Collectively, these results suggest that the connection between gut microbiota and the brain plays a vital role in the occurrence, development, and treatment of gut-brain disorders.

Although several communication pathways contribute to the gut microbiota on brain physiology and behavior, the deep mechanisms by which enteric bacteria communicate with the brain and how the gut microbiota influences the brain causally are still needed to be illustrated. Moreover, further understanding of the roles and mechanisms of psychobiotics in treating various gut-brain disorders may help generate new therapeutic strategies for neural disorders in humans.

This Research Topic brings nine articles summarizing the regulation of intestinal microbes to gut-brain disorders in mammals, the potential mechanisms of targeting microbiota in the treatment of neurological disorders *via* the gut-brain axis, and the beneficial effects of psychobiotics on some neurodegeneration diseases.

In their review article, Chen et al. provide an overview on the current knowledge related to the potential microbial mechanisms and metabolic effects in the progression of Hepatic Encephalopathy (a neurological disorder that occurs in individuals with chronic liver diseases) *via* the perspective of gut-brain axis. They also discuss novel therapeutic strategies (e.g., fecal microbiota transplantation (FMT) and providing specific probiotics) that maintain intestinal homeostasis is vital for treating liver disease-related neurological disorders. Tang et al. extend these concepts to Hippocampus-dependent neurodegenerative diseases. Their review outlines the recent findings on the relationship between intestinal microbes and the hippocampus’s plasticity, neurochemicals, and function. They also highlight the advances in modulating hippocampal structure and behavior using probiotics, prebiotics, and diet through the gut microbiota-hippocampus axis. Cognitive impairment, characterized by conditions where a huge range of mental deficits are expressed, is reported to be an early symptom of the preclinical AD spectrum (Xu et al., 2021). *Porphyromonas gingivalis*, the key periodontal pathogen that could be detected in the brain of AD patients, can induce cognitive impairment and dysbiosis of the gut and then contribute to the occurrence and development of AD (Diaz-Zuniga et al., 2020). As described by Chi et al., *P. gingivalis* infection of oral origin causes the gut microbiota dysbiosis, neuroinflammation, and glymphatic system impairment in mice, resulting in cognitive decline through disturbing the microbiota-gut-brain axis. Moreover, they discuss the potential molecular basis of *P. gingivalis* infection-induced neurological diseases, including how it affects brain functions and disturbs the gut barrier.

Depression following stroke, also known as post-stroke depression (PSD), is a common mood disorder with one symptom of the altered gut microbiome and its metabolites, indicating that gut microbiome may participate in the development of PSD. A study presented in the issue comparing the profile of gut microbiome and fecal metabolomics in rats suffering PSD found that the microbial and metabolic phenotypes were changed significantly in PSD rats (Jiang et al.). Furthermore, the changed gut microbes were highly consistent with their behavioral performance and correlated with the disturbances of fecal metabolomics that mainly assigned to lipid metabolism in PSD rats. Although studies focused on the neurological aspects of stroke that were accumulating in the last decades, researchers should explore deep insights into the relationship between gut microbiota and stroke. In their article, Zhao et al. summarize recent progress in the interactions between gut microbiota and ischemic stroke (the most common stroke events caused by the blockage of blood flow), including how stroke affect gut microbiota composition and how these changes reversely influence stroke outcome and prognosis. Moreover, they discuss that modulating gut microbiota by psychobiotics or prebiotics are helpful in the post-stroke therapy, such as specific functional bacterial species (e.g., *Bifidobacterium longum*), natural products (e.g., *Panax Notoginsenoside* extract) and metabolic compounds (e.g., lactulose). They also suggest that concerning gut microbiota will provide novel avenues to treat post-stroke disorders and remind clinicians for exceptional care in post-stroke management.

Microbiota-based therapeutic strategies have demonstrated the potential to alleviate symptoms of neurological disorders in various preclinical models. As described by Yuan et al., lactulose supplementation has been used to treat neurological outcomes due to its ability to improve neurological function, suppress inflammation in the brain and gut, regulate microbiota and associated metabolic disturbance, and inhibit harmful bacteria *in vivo*. Another study performed by Zhang et al. suggests that *Sophora alopecuroides* L.-derived alkaloids can improve depression-like behaviors and depression-related indicators through modulating gut microbiota, which reveals the mechanism of action of alkaloids in the treatment of brain disorders.

Manipulation of the gut microbiota by FMT is an emerging therapeutic strategy that has been shown to improve cognitive function and brain development disorders (Vendrik et al., 2020). A clinical trial performed by Li et al. suggests that FMT can relieve gastrointestinal and autism symptoms without inducing any severe complications by improving the gut microbiota in children with autism spectrum disorders. Moreover, this study highlights a specific microbiota intervention that targets *Eubacterium coprostanoligenes* to enhance the FMT response. Washed microbiota transplantation (WMT), another version of modified FMT, is demonstrated to be safer, more precise and more quality-controllable than FMT (Zhang et al., 2020). In this issue, Wang et al. report that WMT partially rescues the alterations of behaviors, microbiota composition, and brain structures by light-induced stress.

In conclusion, this Research Topic provides readers with an overview of the potential role of gut microbiota and its metabolic profiles in the occurrence, development, and treatment of associated neurological disorders. However, the precision medicine of gut-brain conditions *via* the microbiota-gut-brain axis remains in the distant future until the physiological and molecular mechanisms underlying these connections could be deeply elucidated. And more clinical researches should be carried out to support the new therapeutic strategies targeting gut microbiota for neural disorders in humans.

## Author Contributions

SL, JW, and TC wrote and revised this article. All authors made a substantial, direct and intellectual contribution to this work, and approved it for publication.

## Funding

This work is supported by grants from the National Natural Science Foundation of China (Grant no. 31560264), Academic and technical leaders of major disciplines in Jiangxi Province (Grant no. 20194BCJ22032), and Double thousand plan of Jiangxi Province (high end Talents Project of scientific and technological innovation).

## Conflict of Interest

The authors declare that the research was conducted in the absence of any commercial or financial relationships that could be construed as a potential conflict of interest.

## Publisher’s Note

All claims expressed in this article are solely those of the authors and do not necessarily represent those of their affiliated organizations, or those of the publisher, the editors and the reviewers. Any product that may be evaluated in this article, or claim that may be made by its manufacturer, is not guaranteed or endorsed by the publisher.
